# Supplementary material for: Integrated analysis of human DNA methylation, gene expression, and genomic variation in iMETHYL database using kernel tensor decomposition-based unsupervised feature extraction
Source: PLoS One. 2023 Aug 9;18(8):e0289029. doi: 10.1371/journal.pone.0289029 (PMC10411815; doi:10.1371/journal.pone.0289029)
Supplement: Attachment — Submitted filename: Replies_to_Reviewers_Iwate_med_PLoSONE.docx [file pone.0289029.s001.docx]

**Replies to reviewer 1:**

Reviewer #1: Comments to the Author
The given manuscript entitled, “Integrated analysis of human DNA methylation, gene expression, and genomic variation in iMETHYL database using kernel tensor decomposition-based unsupervised feature extraction" integrated the gene expression, DNA methylation, and genome variants for 194 subjects; divided as CD4+ T cells (n=99), monocytes (n=99) and neutrophils (n=94) from the iMETHYL database using the kernel tensor decomposition-based unsupervised feature extraction with very limited computational resources. The work comprised the analysis of data sets from these 194 subjects from iMETHYL database. Further, Pre-processing of data-sets comprised of: Fastq files from RNA-Seq were processed through the adapted GTEx pipeline V8; alignment of sequence reads to the GRCH37 human genome using STAR v2.5.0. Also, the sequence reads from whole-genome bisulphite sequencing were aligned using NovoAlignV3.02.08 etc. tensor decomposition-based unsupervised feature extraction were applied and genes associated with selected genomic regions were identified using R. It was followed by enrichment analysis and TF regulation analysis and TFBSs and genes associated with variants were identified.
Based on this background, study results, this reviewer feels that the manuscript writing and methodology adopted needs more clarity and needs certain improvement in the light of following comments and can be accepted after minor revisions for the esteemed journal PLOS ONE.
Minor Criticism:
A. Abstract & title & Aim :
The title should be improved. Word iMETHYL does not convey its true essense . Since iMETHYL is a database, iMETHYL should be replaced with iMETHYL database. The authors should be very careful about the title so that it conveys the true essense of the work presented. The statement, “ Our methods do not even require pre-knowledge about the subjects, as they are fully unsupervised.” Seems ambiguous and perhaps need amendment. In the abstract, statement “Integrating gene expression, DNA methylation, and genomic variants …….is impossible” is contradicting the present research. It is also contradicting the further statement in introduction. Kindly update/clarify.

***> Thank you very much for your comments. We have modified title, abstract on this point.***

B. Introduction:
Kindly make the sentence in agreement with abstract/ here.

***> Thank you very much for your comments. We have modified title and abstract so as to be coincident with introduction.***

C. Materials and Methods:
1. The web-link for iMETHYL database should be provided.

***> Thank you very much for your comments. we have added a link.***

2. Approval no. from ethics committee should be mentioned.

***> Thank you very much for your comments. we have added approval numbers.***

3. Flow diagram (s) of methodology and pipelines used should be provided.

***> Thank you very much for your comments. we have added flow chart. .***

D. Results: well written
E. Discussion: Adequate.
The use of abbreviation for any terminology should be first expanded on its first use.

The authors are encouraged to improve the manuscript for any typographical and grammatical errors by reading the manuscript word by word.

**Replies to reviewer 2:**

Reviewer #2: The authors applied tensor decomposition-based unsupervised feature extraction approach to integrate gene expression, DNA methylation, and genome variants and detect joint features. Multiple applications of the methods were used to demonstrate approach. I have the following comments for the authors to consider:
Major comments are in the section of Material and methods.
1) Material and methods:

a. It will be helpful to explain the method with more detailed information to justify the use of u_l2j to detect features and help readers understand the rationale.

***> Thanks for your comments. We did not use u_l2j_ to select features but used u_l2ik_ (see Eq. (4)). Sorry for easy-to-misleading notations. In addition to this, we stopped using u_l2ik_ but started using u_l1ik_. Since l1 and l2 are mathematically equivalent because of the symmetricity of x_jj’k_ between j and j’, there are no reasons to use l2 instead of l1. The uasege of l2 instead of l1 might have caused confusions.***

It seems u_l2j represents loading?

***> Thanks for your comments. In contrast to the principal component analysis, there are no distinction between loading and score in tensor decomposition. They are just singular value vectors. Sorry for our inappropriate explanations.***

The two references the authors cited are both books without specific indications of the sources of the methods, making the understanding of the approach more challenging.

***> Thank you very much for your comments. Two our previous publications we cited in this manuscript are Refs. 11 and 19. Although Ref. 19 is a book as pointed by the reviewer 2, Ref. 11 is an open access paper that describes almost identical treatment with the present one. While Ref. 11 intergated four omics datasets, methylation, gene expression and two proteme, the present one integrates gene expression, methylation and genomic variatnts. Thus methodology to integrate multiomics data in Ref. 11 is applicable to the present study without almost no modification. Sorry for our bit conusing citations.***

b. The notations of the variables and data are confusing in terms of their dimensionality.
c. The p-value defined in (4) is for testing H0: u_.ik=0. This definition was never used in subsequent analyses and thus unclear about its purpose.

***> Thanks for your comments. Our null hypothesis H0 is not u_l1ik_=0 but u_l1ik_ obeys Gaussian distribution. Sorry for our hard-to-understand description of H0.***

d. In the Introduction section, the authors noted that an existing method did not discuss the relationship between copy number variants and DNA methylation. However, from my understanding, the method applied in the current study was not able to assess the relationship among the three omics either.

***> Thanks for your comments. We believe that we could integrate three omics data using tensor decomposition. As aresults, three omics data share the common sanple dependence represented by u_l2j_, whose biological meaning was detailly analysed in Table 3. Thus we believed that we could successfully relate three omics data sets. Sorry for not-easy-to-understand description of the methodology.***

e. For genetic variants data, since the values are ordinal with three levels, it is unclear if the proposed decomposition still works and does not violate the assumptions.

***> Thanks for your comments. As pointed out by the reviewer 2, it is not guaranteed not to violate the assumption. However, as can be seen in Table 16, our selected variants are signifncatly overlapped with TFBS. Thus. we believed that our assumptions were not broken as a result. Sorry for not explaining this point well.***

2) Results: A lot information is provided in this section. It might be helpful to provide a flow chart of the results to guide readers through.

***>Thanks for your comments. We have provided a flowshart of the results. Sorry for not privinding it in the initial submission.***

Minor comment: Lines 24 to 26 in the subsection “Data Set”, it is unclear how the number 194 was reached. Maybe a Venn diagram will help.

***> Thanks for your comments. We have provided Venn diagrams. We did not mean that 194 subjects were common among three cell types, but common among three measurements (gene expression, DNA methylation and variation) in each of three cell types. Since the number of cell types and mesurements types are both three, there might have been some confusions. In order to avoid the confusion, we have added some more explanation.***

**Replies to reviewer 3:**

Reviewer #3: Integrated analysis of human DNA methylation, gene expression, and genomic
variation in iMETHYL using kernel tensor decomposition-based unsupervised feature extraction by Taguchi et al.

In this article, the authors propose a novel method to be able to integrate multiomic data (DNA methylation, gene expression, and genomic variants) with limited bioinformatics resources despite be non-coincidents (i.e.,the distance between them). They propose a method in which they enrich the data that are connected through regulation by transcription factors. They analyzed data profiles from 194 healthy subjects.
To accomplish with this goal, they used a Tensor decomposition-based unsupervised feature extraction. Authors also explain the step-by-step process to enrich the data and form a biologically significant set.
The manuscript is well written, authors detail each step of the process (i.e. formulas, tables), they have already adjusted the p-values which is a requisite because of the multiple comparisons performed.
I found this paper interesting. I have minor comments:
-This was done with peripheral blood cell data, my question would be if this can be extrapolated, used for other types of cells.

> Thanks for your comments. Yes, our methods is applicable to wide range of type of cells as described in our book (Ref. 19).

-I could not find demographic characteristics of these samples. I understand that this is not the aim of the manuscript, but I was wondering if the authors adjusted the multiomic data for variables like sex and age?. Please comment on this.

***> No. We did not adjusted them at all. As can be seen in the manuscript, our method is fully unsupervised. Thus no labeling (classification) was used to select features at all. Labelng (or classification) was used only when validating u_l2j_ as in Table 3. Thus no adjustment is required.***

-The sample analyzed by the authors was small (n=194), is this method able to analyze datasets of multiomic data from large cohorts of participants?

***>Thanks for your comments. Since the the size of tenser is at most n^2^ K where n is samples numbers and K is the number of omics (K=3), we can deal with much more samples without any difficulties.***

-Regarding statistical significance and p-values, please, comment on the effect size (I mean different effect for methylation, gene expression and genetic variants) which is a critical requirement for multiple testing correction on thousands of genes in these multiomic analyzes.

***> Thanks for your comments. There are no effect size in this analysis, since u_l2j_ is always standardiazed as unit vector (This is inevitable outcome as long as we use HOSVD as tensor decomposition, since HOSVD always gives us unit vector as a result. It is algorithmic requirement). p-values are idnetically related to u_l2j_/sigma_l2_.***

-In table 16, please include the corresponding IC for the OR values.

***> Thanks for your comments. ORs ware already displayed at the lowet rows in individual tables in Table 16. See “odds ratio”.***

-it is recommended that the authors comment if there are limitations in their study.

***> Thanks for your commenyts. We have mentioned the limitations of the method at the end of discussion section.***

Minor misspelling: -in Table 2 “Clincally”

***> Thanks for your commenyts. We have fixed this problem.***
